# Whole-exome sequencing exploration of acquired uniparental disomies in B-cell precursor acute lymphoblastic leukemia

**DOI:** 10.1038/s41375-018-0191-0

**Published:** 2018-07-02

**Authors:** Kristina B. Lundin-Ström, Andrea Biloglav, Henrik Lilljebjörn, Marianne Rissler, Thoas Fioretos, Markus Hansson, Mikael Behrendtz, Anders Castor, Linda Olsson, Bertil Johansson

**Affiliations:** 1 Division of Clinical Genetics, Department of Laboratory Medicine, Lund University, Lund, Sweden; 2 Department of Clinical Genetics and Pathology, Division of Laboratory Medicine, Lund, Sweden; 3 Division of Hematology, Skåne University Hospital and Wallenberg Centre for Molecular Medicine, Lund University, Lund, Sweden; 4 Department of Pediatrics, Linköping University Hospital, Linköping, Sweden; 5 Department of Pediatrics, Skåne University Hospital, Lund, Sweden

Acquired whole chromosome/segmental uniparental disomies (wUPDs/sUPDs) are common in myeloid malignancies [1, 2]. Already in the first publication on acquired UPDs in AML [1], it was reported that a case with UPD19q12-qter harbored a homozygous mutation in the *CEBPA* gene in 19q13.11 and, soon afterward, AML cases with sUPDs of 11p, 13q, and 21q were shown to carry homozygous mutations of the *WT1*, *FLT3*, and *RUNX1* genes [3]. There are now several examples of other UPD-associated homozygously mutated genes in myeloid malignancies, where heterozygous somatic mutations precede the mitotic recombination events [2].

UPDs occur with a similar frequency in BCP ALL as in AML (~20%) [4, 5]. However, next to nothing is known about the molecular consequences of UPDs in BCP ALL. UPD9p is the only UPD investigated in any detail in BCP ALL, where it is often associated with homozygous *CDKN2A* deletions [4], and, to the best of our knowledge, UPD16p is the only UPD in BCP ALL that has been recurrently shown to result in homozygosity of a mutated gene (*CREBBP* at 16p13.3) [6]. To ascertain whether UPDs in BCP ALL are associated with the presence of homozygous gene variants within the affected regions, we performed whole-exome sequencing (WES) analyses of 25 UPD-positive BCP ALL cases.

The acquired wUPDs and sUPDs were identified by single-nucleotide polymorphism array analyses of paired diagnostic/remission samples at the Department of Clinical Genetics and Pathology, Lund, Sweden, as previously reported [4]. The 25 cases comprised 10 with high hyperdiploidy (HeH; 51–67 chromosomes), 3 *ETV6*-*RUNX1*, 2 *BCR*-*ABL1*, 2 *TCF3*-*PBX1*, 1 with *KMT2A* rearrangement, and 7 with non-characteristic abnormalities/normal karyotypes (B-other), with a total of 16 wUPDs and 21 sUPDs. The wUPDs involved chromosomes X, 1, 3, 5, 7, 8, 9, 11, 13, 16, and 19 and all but one were found in the HeH subgroup. None of the wUPDs was associated with a homozygous deletion. The sUPDs occurred in all genetic subgroups and affected chromosome arms 6p, 7p, 9p, 9q, 10q, 11q, 12p, 12q, 14q, 16p, 17p, 17q, 18q, and 19p. Four cases with UPD9p had homozygous *CDKN2A* deletions (Supplementary Table 1).

WES was performed, as described in Supplementary Materials and Methods, on DNA extracted from bone marrow/peripheral blood cells obtained at diagnosis and during remission. The bioinformatic analyses focused on homozygous variants in the leukemic samples that were homozygously wild type in the paired remission samples, revealing a total of 373 putative somatic homozygous variants in 24/37 (65%) UPDs (10 wUPDs and 14 sUPDs) in 15/25 (60%) cases (#1, 4–6, 8–14, 17, and 21–23; Supplementary Table 1). Although UPD16p has been shown to result in homozygosity of *CREBBP* in BCP ALL [6], case 1 with sUPD16p did not harbor any variants in this gene.

Among the 373 variants, the 173 (46%) intragenic splice-site or exonic stop lost, deletions/insertions (delins), or non-synonymous missense variants were selected for further analysis (Fig. 1). Forty-one variants in different *HLA* genes were excluded due to the highly polymorphic nature of these genes and the technical and bioinformatic challenges they present. This resulted in 132 candidate variants in eight cases, of which 128 (97%) were associated with sUPDs involving 6p, 9p, 11q, 12q, and 19p and four (3%) with wUPDs of chromosomes 3 and 11 (cases 4, 5, 8, 11–13, 21, and 22; Supplementary Table 1). Sixty-five of the variants in 55 genes in six cases were confirmed, by Sanger sequencing (Supplementary Materials and Methods; Supplementary Tables 2 and 3), to be acquired (cases 4, 5, 12, 13, 21, and 22) (Fig. 1). The remaining variants could not be verified because of: (i) lack of DNA (59 variants; Supplementary Table 4); (ii) failed sequencing reactions (2 variants); (iii) six variants were not homozygous at diagnosis and wild type at remission.

**Fig. 1:**
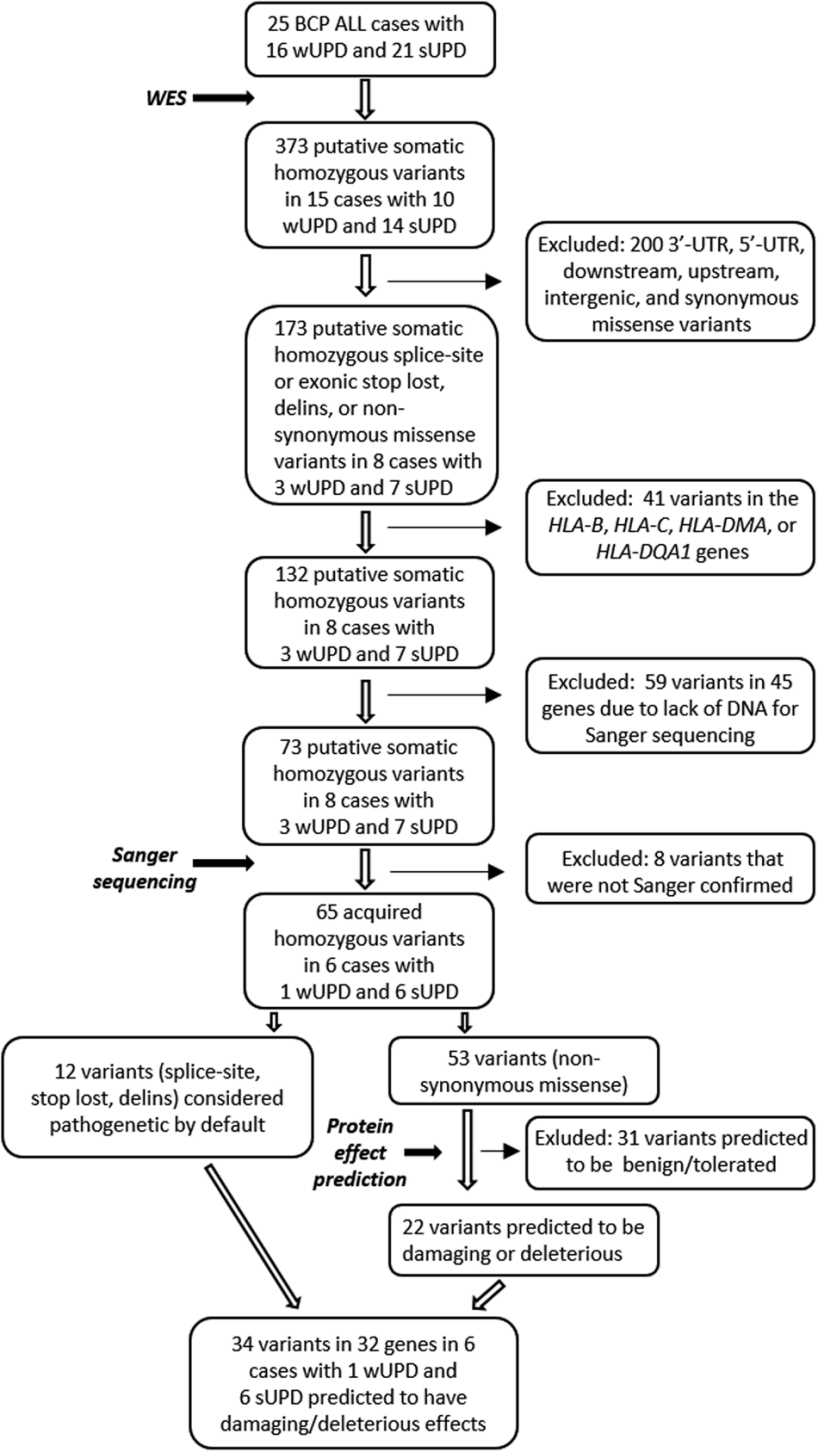
Flowchart of the number of cases and wUPDs/sUPDs investigated in the various analysis steps

The PolyPhen (http://genetics.bwh.harvard.edu/pph2/), PROVEAN (http://provean.jcvi.org/index.php), and SIFT (http://sift.jcvi.org/) software tools were used to predict whether the 65 Sanger-confirmed variants affected protein function. The 12 splice-site, stop lost, and delins were considered pathogenetic by default. Of the 53 non-synonymous missense variants, 22 were predicted to be “possibly damaging”, “probably damaging”, “damaging”, or “deleterious” by at least one of the three programs. In total, 34 UPD-associated acquired homozygous splice-site, stop lost, delins, and non-synonymous missense variants in 32 genes were predicted to have damaging/deleterious effects on their corresponding proteins (Fig. 1, Table 1). The variants occurred in 6 (24%) of the 25 cases (4/7 (57%) B-other cases, 1/2 (50%) *BCR*-*ABL1*-positive cases, and 1/10 (10%) HeH cases), and were found in 7 (19%) of the 37 UPDs (1/16 (6%) wUPDs and 6/21 (29%) sUPDs; cases 4, 5, 12, 13, 21, and 22; Table 1). Six variants (in the *ATG7*, *DNAJC13*, *JAK2*, *SH2B3*, *UBAP2*, and *ZSCAN23* genes) were not listed in dbSNP Build 141 (https://www.ncbi.nlm.nih.gov/projects/SNP/snp_summary.cgi?build_id=141), whereas the remaining 28 damaging/deleterious gene variants have all been reported as germline variants in dbSNP Build 141. However, they were clearly somatic in our cases, not seen in matched remission samples.

**Table 1:** The 34 UPD-associated somatic homozygous variants in 32 genes predicted to have damaging/deleterious effects

Case no.	UPD	Gene symbol^a^	Genetic subgroup	Type of variant	Exon no.	Protein position	Amino-acid change	Software tool		
								PROVEAN	SIFT	PolyPhen
4	11q	***CBL***	B-other	Delins	9	460–461	Deletion of Asp	—	—	—
5	12q	*GRIP1*	B-other	Missense	3	84	Arg → Gln	Deleterious	Damaging	Probably damaging
5	12q	***SH2B3***	*“*	Delins	6	396	Premature stop	—	—	—
12	9p	***JAK2***	B-other (DS)	Missense	16	683	Arg → Gly	Deleterious	Damaging	Probably damaging
13	9p	*CER1*	B-other	Missense	1	19	Arg → Trp	Neutral	Damaging	Benign
13	9p	***FREM1***	*“*	Missense	10	465	Gly → Ala	Deleterious	Damaging	Probably damaging
13	9p	*CNTLN*	*“*	Missense	11	562	Arg → Cys	Deleterious	Damaging	Benign
13	9p	*“*	*“*	Splice-site	—	—	—	—	—	—
13	9p	*IFT74*	*“*	Missense	20	597	Thr → Ile	Neutral	Damaging	Benign
13	9p	*IFNK*	*“*	Delins	1	13	Premature stop	—	—	—
13	9p	*DDX58*	*“*	Missense	1	7	Arg → Cys	Deleterious	Damaging	Probably damaging
13	9p	*UBAP2*	*“*	Missense	14	509	Arg → Trp	Deleterious	Damaging	Probably damaging
21	6p	*HUS1B*	*BCR-ABL1*	Missense	1	201	Gln → Arg	Neutral	Damaging	Benign
21	6p	***BMP6***	*“*	Missense	1	96	Leu → Pro	Neutral	Tolerated	Possibly damaging
21	6p	*ATXN1*	*“*	Missense	8	753	Pro → Ser	Neutral	Damaging	Benign
21	6p	*ZSCAN23*	*“*	Splice-site	—	—	—	—	—	—
21	6p	*OR2W1*	*“*	Missense	1	81	Met → Val	Neutral	Damaging	Benign
21	6p	*UBD*	*“*	Missense	2	68	Ile → Thr	Deleterious	Damaging	Probably damaging
21	6p	*GTF2H4*	*“*	Splice-site	—	—	—	—	—	—
21	6p	*CDSN*	*“*	Missense	2	410	Leu → Ser	Neutral	Tolerated	Possibly damaging
21	6p	*STK19*	*“*	Splice-site	—	—	—	—	—	—
21	6p	*NOTCH4*	*“*	Delins	1	15	Deletion of Ser	—	—	—
21	6p	*TAP2*	*“*	Stop lost	12	687	Stop → Gln	—	—	—
21	6p	*RPL10A*	*“*	Splice-site	—	—	—	—	—	—
21	6p	*PNPLA1*	*“*	Missense	6	193	Glu → Gly	Deleterious	Damaging	Possibly damaging
21	6p	*PEX6*	*“*	Missense	13	809	Ala → Val	Deleterious	Damaging	Probably damaging
21	19p	*TMIGD2*	*“*	Missense	3	138	Pro → Leu	Deleterious	Tolerated	Benign
21	19p	*SAFB2*	*“*	Missense	10	457	Thr → Ala	Deleterious	Damaging	Benign
21	19p	*FCER2*	*“*	Splice-site	—	—	—	—	—	—
21	19p	*“*	*“*	Splice-site	—	—	—	—	—	—
21	19p	*RGL3*	*“*	Missense	5	162	Pro → His	Deleterious	Damaging	Probably damaging
21	19p	*PALM3*	*“*	Missense	6	412	Thr → Arg	Neutral	Damaging	Possibly damaging
22	3	*ATG7*	HeH	Missense	3	62	Arg → His	Deleterious	Damaging	Probably damaging
22	3	*DNAJC13*	*“*	Missense	52	2039	Pro → Ser	Deleterious	Damaging	Benign

*DS* Down syndrome, *HeH* high hyperdiploidy (51–67 chromosomes), *UPD* uniparental disomy

^a^Genes previously reported to be involved in B-cell precursor acute lymphoblastic leukemia are indicated in bold type

The present study is the first to focus specifically on the possible association between UPDs and homozygous gene mutations in BCP ALL. WES analysis of UPD-positive BCP ALL cases identified a large number of candidate variants—most of which are probably “passengers”. It is, however, noteworthy that 97% of the 132 candidate variants were associated with sUPDs and only 3% with wUPDs. This strongly indicates that the pathogenetic consequences of sUPDs and wUPDs differ. We have previously shown that wUPIDs in pediatric BCP ALL are restricted to the HeH subgroup, where they may reflect the underlying mechanism of the high hyperdiploid pattern in some cases, namely an initial tetraploidization followed by chromosome losses. This would result in a third of the disomies being wUPDs by chance alone [4]. Because the numerical abnormalities in HeH ALL are early, likely primary, events in the genesis of this subtype [7], the present finding of a general lack of homozygous variants in wUPDs may simply reflect that variants most often arise after wUPD formation and hence are heterozygous. It is, however, noteworthy that two homozygous variants associated with wUPD3 were identified in case 22; thus, they occurred prior to the wUPD event. The sUPDs, on the other hand, are probably later events in the leukemogenic process and hence more likely to duplicate somatically acquired variants.

The final set of 34 variants were observed in only 24% of the 25 BCP ALL cases and 19% of the 37 UPDs, but were enriched in B-other cases and in sUPDs (Table 1). This genetically and clinically heterogeneous group has received much attention in recent years, with the aim to subdivide it into homogeneous subtypes, on the basis of gene expression profiles and patterns of fusion genes, deletions, and mutations. As the present study shows, WES analyses of sUPDs in B-other cases may provide pertinent data in this regard by identifying genes of potential pathogenetic importance in this subtype.

None of the 32 genes targeted by the 34 variants was recurrently involved among the cases analyzed. Furthermore, only five of the targeted genes have previously been implicated in BCP ALL. *BMP6* and *FREM1* mutations have so far been reported in single BCP ALL cases only (https://cancer.sanger.ac.uk/cosmic), whereas *CBL*, *JAK2*, and *SH2B3* mutations are recurrent in BCP ALL. *CBL* is rarely mutated in BCP ALL, but identifying cases with *CBL* mutations may be clinically relevant because they have been shown to be associated with constitutive activation of the RAS pathway and to be sensitive to MEK inhibitors [8]. *JAK2* mutations, resulting in constitutive activation of the JAK-STAT signaling pathway, are present in ~4% of BCP ALL in general but are particularly common (~20%) in BCP ALL in children with Down syndrome (DS) [9]. The single case with UPD9p and a homozygous *JAK2* mutation in our cohort was also a DS-ALL (Table 1). In addition, this case had a homozygous *CDKN2A* deletion associated with the UPD9p. This shows that a sUPD can result in homozygosity of two distinct gene changes; thus, one should not assume that UPD9p-associated homozygous *CDKN2A* deletions are the only pathogenetically important consequences of this sUPD. *SH2B3* plays an important role in the homeostasis of hematopoietic stem cells and lymphoid progenitors, and homozygous somatic *SH2B3* mutations have previously been identified in ALL, suggesting a tumor suppressor role [10]. The underlying mechanism for the homozygosity of *SH2B3* mutations has not been previously addressed, but, as seen herein, UPD12q is clearly one mechanism.

Among the 27 novel BCP ALL-associated genes identified, five have previously been implicated in other hematologic malignancies: *DDX58* regulates proliferation of AML cells [11], *ATXN1* and *TAP2* polymorphisms are associated with increased risks of chronic lymphocytic leukemia and follicular lymphoma, respectively [12, 13], and deletion of *Atg7* in a murine model results in a myeloproliferation resembling human AML [14]. Furthermore, *NOTCH4* activation has been shown to lead to reduced differentiation and altered lymphoid development [15]. Thus, these genes may be of particular interest in BCP ALL. However, this remains to be elucidated, as does the potential impact of the other gene variants (Table 1).

In summary, we conclude that sUPDs in BCP ALL may highlight chromosomal regions carrying genes of importance in the leukemic process, particularly in the B-other group.

## Electronic supplementary material

Supplementary Materials and Methods

Supplementary Table 1

Supplementary Table 2

Supplementary Table 3

Supplementary Table 4
