# Supplementary material for: Whole-exome sequencing exploration of acquired uniparental disomies in B-cell precursor acute lymphoblastic leukemia
Source: Leukemia. 2018 Jul 2;32(9):2058–62. doi: 10.1038/s41375-018-0191-0 (PMC6127080; doi:10.1038/s41375-018-0191-0)
Supplement: Supplementary file 1 — Supplementary Materials and Methods [file 41375_2018_191_MOESM1_ESM.docx]

**SUPPLEMENTARY MATERIALS AND METHODS**

Exon enrichment and whole exome sequencing

DNA, extracted using standard methods from bone marrow/peripheral blood cells obtained at diagnosis and during remission, was enriched for coding exons with the TruSeq Exome Enrichment kit (Illumina, San Diego, CA, USA; cases 1-5) or with the Nextera Rapid Capture Exome kit (Illumina; cases 6-25) according to the manufacturer’s protocols. The integrity and the fragment sizes of the DNA were confirmed on a 2100 Bioanalyzer (Agilent, Santa Clara, CA, USA).

The libraries from cases 1-5 were analyzed on an Illumina HiScan SQ, generating paired-end 2x100 base pair reads (cases 1-3 were analyzed with a coverage of ~60X and cases 4 and 5 with a coverage of 100X). The libraries from cases 6-25 were analyzed on an Illumina NextSeq 500, generating paired-end 2x150 base pair reads with a coverage of 130-150X.

Data analyses

The data analyses were performed in the Strand NGS software, Version 2.6, Build 230243 (Strand Life Sciences, Bangalore, India), according to the Strand NGS manual for Version v2.6. In cases 1-5, the Fastq files were aligned to the GRCh37 build of the human genome using the Burrows-Wheeler Alignment tool.^1^ PCR duplicates were removed using Picard (http://broadinstitute.github.io/picard/) before uploading the resulting BAM files to Strand NGS. In cases 6-25, the Fastq files were uploaded directly to Strand NGS and aligned to the GRCh37 build, including an adaptor-trimming step. Post-Alignment QC and Targeted Region QC were performed and calculated for all cases. Reads with inconsistent mate status were removed, after which local realignment and base quality recalibration were performed. Reads were further filtered on duplicates and read status. SNP detection was performed on the whole exome and annotated to dbSNP Build 141 (https://www.ncbi.nlm.nih.gov/projects/SNP/snp_summary.cgi?build_id=141). The cases were then analyzed in the Strand NGS software for somatic variants and their genetic consequences (SNP effect). The data were further filtered in Microsoft Excel to cover only the respective UPD regions in each case, focusing on genes with homozygous variants in the leukemic samples that were homozygously wild type in the paired remission samples. Approximately 70% supporting reads in the diagnostic samples in combination with <1% supporting reads in the remission samples were used as a cut-off for putative acquired UPD-associated homozygous variants. Candidate variants were visually inspected and compared between diagnostic and remission samples in the Strand NGS Genome Browser.

Verification by Sanger sequencing

Primers for PCR amplification and Sanger sequencing of the gene regions with candidate variants were designed in Primer3Plus (http://primer3plus.com/cgi-bin/dev/primer3plus.cgi). Primer sequences used for the subsequently verified somatic homozygous variants are given in Supplementary Table 2. The amplified DNA was sequenced using the BigDye Terminator Cycle Sequencing Kit (Life Technologies, Carlsbad, CA, USA) and analyzed on an ABI 3130 Genetic Analyzer (Life Technologies). Variants were identified in Chromas v2.6.2 (Technelysium Pty Ltd, South Brisbane, Australia).

REFERENCE

1 Li H, Durbin R. Fast and accurate short read alignment with Burrows-Wheeler transform. *Bioinformatics* 2009; **25**: 1754-1760.
