# Supplementary material for: Whole-exome sequencing exploration of acquired uniparental disomies in B-cell precursor acute lymphoblastic leukemia
Source: Leukemia. 2018 Jul 2;32(9):2058–62. doi: 10.1038/s41375-018-0191-0 (PMC6127080; doi:10.1038/s41375-018-0191-0)
Supplement: Supplementary file 2 — Supplementary Table 1 [file 41375_2018_191_MOESM2_ESM.docx]

**Supplementary Table 1.** The 25 BCP ALL cases with acquired wUPDs/sUPDs analyzed by WES

| *Case* | *Sex/* | *WBC* | *Genetic* | *wUPD* | *sUPD* | *sUPD position* |
| --- | --- | --- | --- | --- | --- | --- |
| *No.* | *age* | *(x10^9^/l)* | *subgroup* |  |  | *GRCh37* |
| 1 | M/5 | 16 | HeH | — | 16p11.2-pter | Chr16:pter-28218704 |
| 2 | F/3 | 30 | *ETV6*-*RUNX1* | — | 18q21.32-qter | Chr18:57207198-qter |
| 3 | F/10 | 2.6 | *ETV6*-*RUNX1* | — | 9p13.2-pter | Chr9:pter-36744730 |
| 4 | M/0 | 4.3 | B-other | — | 11q13-qter | Chr11:62265585-qter |
| 5 | M/15 | 59 | B-other | — | 7p22.3-pter | Chr7:pter-2130014 |
|  |  |  |  |  | 12q14.1-qter | Chr12:62691991-qter |
| 6 | M/3 | 3.1 | *ETV6*-*RUNX1* | — | 12p13.2-pter | Chr12:pter-11708953 |
| 7 | M/13 | 66 | HeH | 9 | 17p11.2-13.2 | Chr17:5550299-18291618 |
| 8 | F/8 | 5.0 | HeH | 3, 19 | 10q23.1-25.2 | Chr10:86058516-112294424 |
| 9 | M/1 | 30 | HeH | 8 | — |  |
| 10 | M/10 | 4.1 | HeH | — | 9q12-qter | Chr9:70984372-qter |
| 11 | F/2 | 9.7 | HeH | 5 | 9p11.2-pter | Chr9:pter-38771460 |
| 12^a^ | F/2 | 35 | B-other (DS) | — | 9p21.2-pter | Chr9:pter-29470565 |
| 13^a^ | M/10 | 3.2 | B-other | — | 9p13.3-pter | Chr9:pter-34087360 |
| 14 | M/1 | 55 | *TCF3*-*PBX1* | — | 17q11.2-qter | Chr17:30625767-qter |
| 15 | F/4 | NK | *TCF3*-*PBX1* | — | 14q12-qter | Chr14:25510923-qter |
| 16 | F/5 | 6.3 | HeH | X | — |  |
| 17 | M/2 | 6.2 | HeH | 1, 3, 13, 19 | — |  |
| 18 | M/2 | 24 | *KMT2A* | — | 9p21.1-21.3 | Chr9:20464928-32007381 |
| 19 | M/3 | 14 | HeH | 1, 7, 11 | — |  |
| 20 | M/16 | 13 | B-other (DS) | — | 14q12-qter | Chr14:31188832-qter |
| 21 | M/54 | 122 | *BCR*-*ABL1* | — | 6p21.1-pter | Chr6:pter-43019435 |
|  |  |  |  |  | 19p12-pter | Chr19:pter-21233406 |
| 22 | M/47 | 1.2 | HeH | 3, 11 | — |  |
| 23^a^ | F/23 | 5.5 | B-other | — | 9p13.2-pter | Chr9:pter-36805874 |
| 24 | M/32 | 15 | *BCR*-*ABL1* | 16 | — |  |
| 25^a^ | M/19 | NK | B-other | — | 9p11.2-pter | Chr9:pter-39217322 |

BCP ALL, B-cell precursor acute lymphoblastic leukemia; B-other, non-characteristic changes; DS, Down syndrome; F, female;

HeH, high hyperdiploidy (51-67 chromosomes); M, male; NK, not known; sUPD, segmental uniparental disomy; WBC, white blood

cell count; WES, whole exome sequencing; wUPD, whole chromosome uniparental disomy.

^a^These cases harbored homozygous *CDKN2A* deletions within the UPD9p segments.
