# Supplementary material for: Whole-exome sequencing exploration of acquired uniparental disomies in B-cell precursor acute lymphoblastic leukemia
Source: Leukemia. 2018 Jul 2;32(9):2058–62. doi: 10.1038/s41375-018-0191-0 (PMC6127080; doi:10.1038/s41375-018-0191-0)
Supplement: Supplementary file 3 — Supplementary Table 2 [file 41375_2018_191_MOESM3_ESM.docx]

**Supplementary Table 2.** Primer sequences used for the Sanger sequencing-verified UPD-associated acquired homozygous variants

| *Gene* | *Gene name* | *Start* | *Reference* | *Variant* | *Forward primer (5’→3’)* | *Reverse primer (5’→3’)* |
| --- | --- | --- | --- | --- | --- | --- |
| *symbol* |  | *(GRCh37)* | *allele* | *allele* |  |  |
| *APC2* | APC2, WNT signaling pathway regulator | 19:1467684 | C | G | CACGCCGGTCAACTTCTCTA | TGCCGTAGAAGCAGTACACG |
| *ATG7* | Autophagy related 7 | 3:11340860 | G | A | GTCACATTCTGCCATTTATCTATCCA | TCCAGCACTCATCTCTATGGGA |
| *ATXN1* | Ataxin 1 | 6:16306751 | G | A | TTGCCTACATTAGACCGGCC | CTGAAGCACTCAAAGGCCGA |
| *BMP6* | Bone morphogenetic protein 6 | 6:7727475 | T | C | GTCCTCCTCGGGCTTCCT | CCAGGACTGCTGCCTCTC |
| *CBL* | CBL proto-oncogene | 11:119149355 | TATG | T | TCTTTTGCTTCTTCTGCAGGA | TGGATATCGTTAAGTGTTTTACGGC |
| *CDSN* | Corneodesmosin | 6:31084163 | A | G | CCCAGTCAGTGTCAAGGAGG | CCATTGCATTCCAGCCAGTG |
| *CER1* | Cerberus 1, DAN family BMP antagonist | 9:14720357 | C | T | GTGCAGTTCAGTGGCAAGTG | GGTGGTTCTGTGCCCTTATAGA |
| *"* | *"* | 9:14722616 | G | A | TGCGACAAACAGATCTGGCT | CTACTGACCACCTGCCTTCC |
| *CNTLN* | Centlein | 9:17340864 | C | T | ACAACAGGGAAAGGAGTGAAAC | ACTGCCTTCTTCCCACTGTT |
| *"* | *"* | 9:17486984 | C | T | TGAGATGACAGACTGTTCTTTGT | TCCATTCCTTGTCATTTTCAGCA |
| *DDX58* | DExD/H-box helicase 58 | 9:32526146 | G | A | GTCCTCAATTGTTTTCCGCCG | AAGTTCCTATGCAGCTCCGC |
| *DNAJC13* | DnaJ heat shock protein family (Hsp40) member C13 | 3:132244508 | C | T | TGAATCCCTAGTTTTGAACACCA | ATCCGTCAAGAAGGGAGCAT |
| *ELL* | Elongation factor for RNA polymerase II | 19:18562438 | C | T | GAGCTGGTGAGGAAGGTGAC | CCTGCTCTGGATTTGGGAGG |
| *FCER2* | Fc fragment of IgE receptor II | 19:7755159 | A | G | CCCTCCTCACCTGTAGTCCA | CGGTATGCCTGTGACGACAT |
| *"* | *"* | 19:7755285 | A | G | CCCTCCTCACCTGTAGTCCA | CGGTATGCCTGTGACGACAT |
| *FOCAD* | Focadhesin | 9:20953049 | A | G | TCCTGCCAAGTTCACTGGTT | AGGCATGCTTAGGGTACACA |
| *FREM1* | FRAS1 related extracellular matrix 1 | 9:14842658 | C | G | ATAGCGAACAACTCCAGCCT | AATACGGGCTGCACACTTTC |
| *GPX6* | Glutathione peroxidase 6 | 6:28483482 | A | C | GGGCTGGGAATGCAGATCTT | AATGCCACTCTGCGATTGGA |
| *GRIP1* | Glutamate receptor interacting protein 1 | 12:66935616 | C | T | GACCTGGGGAACAGCTTCTG | ATTCAAGGGCTCCACAGTCG |
| *GTF2H4* | General transcription factor IIH subunit 4 | 6:30880097 | T | C | GTATGCCTACACGGGTGAGG | TGTCTTCCTGCTCCACCTCT |
| *HFE* | Hemochromatosis | 6:26091179 | C | G | AAGGCCTGTTGCTCTGTCTC | CCCTCTCCACATACCCTTGC |
| *HUS1B* | HUS1 checkpoint clamp component B | 6:656343 | T | C | AGGGCCGTCGTAGGATGTAT | GAGAGTGTGGCGGGACTG |
| *IFNK* | Interferon kappa | 9:27524364 | G | GTGTT | GCCGCAACCTTGGTTAACTG | TGCAGAAACTCTTGGGGCAA |
| *IFT74* | Intraflagellar transport 74 | 9:27062721 | C | T | AGTTCCTTTCTCAGGCACAAT | TCATTCTTAAAAGAGGCCACAAAA |
| *JAK2* | Janus kinase 2 | 9:5078360 | A | G | TCTCAATGCATGCCTCCAAA | CCCTTTACACCACTGCCCAA |
| *JAK3* | Janus kinase 3 | 19:17949108 | C | T | GGGGGTTCCTGCAGGATC | CTCAGGGTCGGCTTCTAGAG |
| *KANK1* | KN motif and ankyrin repeat domains 1 | 9:712156 | T | G | GGTGCCGAGGAGAACATGAA | CTGCCTCCACCACCTTACTG |
| *"* | *"* | 9:732536 | T | C | GAGGAGGAGGAGGAGGATGA | GGCCAACTCTATTGCCAAAGT |
| *MBD3L4* | Methyl-CpG binding domain protein 3 like 4 | 19:7038129 | C | T | GCCTGTCTCCGGATATCTGC | TCAGACGCAGAAAAGGGGAC |
| *MTAP* | Methylthioadenosine phosphorylase | 9:21816758 | G | A | TCAGATCTTGCCTCTTCTCTAAGT | AGATGTTCTGATTCTGCCAACA |
| *MUC16* | Mucin 16, cell surface associated | 19:9076278 | G | A | CTGGTGTGACTGCACTTGTA | CAGTACAAGTGAAAGAACATTGAGT |
| *MUC22* | Mucin 22 | 6:31002452 | A | G | ACATGGGCAGTTTGGAAAAGTG | CCATGGCCCACTCCATATCC |
| *"* | *"* | 6:31002527 | A | G | ACATGGGCAGTTTGGAAAAGTG | CCATGGCCCACTCCATATCC |
| *NKAPL* | NFKB activating protein like | 6:28227436 | A | G | CCCGCAGAGCAGATGTTCC | GATGCGTTACCTCTTCTTCATCC |
| *"* | *"* | 6:28227604 | C | A | GGCAGTCAGCGGAAGACTAC | TGTCAGACTCTGAGTTACTGTCAC |
| *"* | *"* | 6:28228342 | A | G | ATGAGTGGTAGCAGGCATCG | AGAAGCTTTTTCCTGAGGGACTT |
| *NOTCH4* | Notch 4 | 6:32188640 | T | C | GGGTGTCATGGATGTGGCTT | GCCAGCTGATGCCAGAGAAA |
| *"* | *"* | 6:32191658 | TAGC^a^ | T | CTCCATCCAGCATCCCTCAC | AGGCCGAGGAGGAAGAAGAG |
| *OR2W1* | Olfactory receptor family 2 subfamily W member 1 | 6:29012712 | T | C | AGAAGGCACTCAACTGAGCC | TGCTTGGCTTCTCTAACCATCC |
| *PALM3* | Paralemmin 3 | 19:14165204 | G | C | TCCCTCTCTGTTCCCAGCTT | ACCCTCAGTGAAGAGTGGGA |
| *PEX6* | Peroxisomal biogenesis factor 6 | 6:42933464 | G | A | GGCCCCTTTCAGCTTCCATT | GTTTCCCTCCCTGCTCTCAC |
| *PGBD1* | PiggyBac transposable element derived 1 | 6:28269407 | G | A | CCTCTTGTATGCTCCCCTGG | GCCCTCACCCCCTTCTTTTT |
| *PNPLA1* | Patatin like phospholipase domain containing 1 | 6:36269725 | A | G | AAGACCTTTGAAAAGCACTGTTCC | CAGGTGGCTGCTCAAGTGG |
| *"* | *"* | 6:36274153 | C | T | CAAGAGAGTTCTTTGCTGTTTTTAAAC | TTCCCCACACACTGCCCTAA |
| *POM121L2* | POM121 transmembrane nucleoporin like 2 | 6:27277051 | C | T | GGAATGCCCTTTCTCCCGAT | TTGGAGCATTGCCTAGTGGG |
| *"* | *"* | 6:27279774 | C | T | GATGGTCACTGGGCTCCAAA | TGGGCAGTTTCCTGAGCAAA |
| *RASAL3* | RAS protein activator like 3 | 19:15574917 | G | A | GACAAAACCCAAGCATCCGG | CTGACTTCCTACCGCTGGC |
| *RGL3* | Ral guanine nucleotide dissociation stimulator like 3 | 19:11526765 | G | T | GGCCTTCTCAGAACACGACT | TCTATGTCCCCCACTCTGGA |
| *RPL10A* | Ribosomal protein L10a | 6:35436571 | C | T | CAGGTCCGATCACTGAGAGC | GGATCATAGTTCTTCAAGCTGATCTG |
| *RRP36* | Ribosomal RNA processing 36 | 6:42992825 | C | G | TTTCTGCAGTGGGACATGCT | GAGGACTCCAGTGTTGGTCA |
| *SAFB2* | Scaffold attachment factor B2 | 19:5604875 | T | C | CCCAGAGCTTCTCACTTGCA | ACCATATGGGGAGAGGTGACA |
| *SH2B3* | SH2B adaptor protein 3 | 12:111885299 | GCG | GAGGG | GTACGCTGGAACCCAGACTC | TGTCCCTCTAGGACCCTGAA |
| *SKIV2L* | Ski2 like RNA helicase | 6:31929014 | A | C | AGTGGGGTGTAGGAGAAGTCA | TGCCTCCACTACACAGAACC |
| *SLC17A3* | Solute carrier family 17 member 3 | 6:25850845 | C | T | AGGGAAGGAAACATACTCACGT | TCCTCCTTGAAACAACAGGTACA |
| *SLC26A8* | Solute carrier family 26 member 8 | 6:35980121 | C | T | TCATGTAGCACAAGTAATTGTTTACCC | AATGCACAGGACAGCCCAA |
| *STK19* | Serine/threonine kinase 19 | 6:31946792 | G | A | CAAAATCTCCCTGCCTGAGAG | CTCCCCCTCCTCTCCCCC |
| *TAP2* | Transporter 2, ATP binding cassette subfamily B member | 6:32796685 | A | G | CAGGAACAGCTATCTGGCCG | GCACTGTGATCACCCCTTCA |
| *TEK* | TEK receptor tyrosine kinase | 9:27190655 | G | A | GCCCCAAACGTGATTGACAC | GATTGAGGCAGGGAGAAGGG |
| *TMIGD2* | Transmembrane and immunoglobulin domain containing 2 | 19:4294807 | G | A | GATCGCAGCCACACCCAT | GCCCAAATCCACAAAGCCTG |
| *TREML2* | Triggering receptor expressed on myeloid cells like 2 | 6:41162518 | T | C | CTGTCTTGGAGGCAGGTCTG | TAGGCCCAAGGTCAAGGAGA |
| *UBAP2* | Ubiquitin associated protein 2 | 9:33944383 | G | A | TCCAGCTTTAGGACGGTGAC | CCTTCCCAGGCAAAACTTCG |
| *UBD* | Ubiquitin D | 6:29523952 | A | G | GAGGTGCCTCTTTGCCTCAT | TGTGGCTTTCACTTCCCAGG |
| *ZNF165* | Zinc finger protein 165 | 6:28056646 | T | G | GCAGGCAGGGTAAAGAGACA | TCTCCACTGAAAACTGCTGCA |
| *ZNF311* | Zinc finger protein 311 | 6:28963248 | T | G | TCTTTTGTGCTGCCTCAGGA | AGGTGTGAGGAGTGTGGGAA |
| *ZSCAN23* | Zinc finger and SCAN domain containing 23 | 6:28403388 | G | A | AAATATTCAGGGAATGAGGTAGGCT | GGGTTTCAGTGCAACAAGAAA |

UPD, uniparental disomy.

^a^Deletion of six AGC repeats; from 12 in the remission sample to six in the diagnostic sample.
