# Supplementary material for: Whole-exome sequencing exploration of acquired uniparental disomies in B-cell precursor acute lymphoblastic leukemia
Source: Leukemia. 2018 Jul 2;32(9):2058–62. doi: 10.1038/s41375-018-0191-0 (PMC6127080; doi:10.1038/s41375-018-0191-0)
Supplement: Supplementary file 4 — Supplementary Table 3 [file 41375_2018_191_MOESM4_ESM.docx]

**Supplementary Table 3.** The 55 UPD-associated genes confirmed by Sanger sequencing to harbor 65 acquired homozygous variants

| *Case* | *Chr* | *Start* | *Reference* | *Variant* | *Gene* | *Gene name* |
| --- | --- | --- | --- | --- | --- | --- |
| *No.* | *No.* | *(GRCh37)* | *allele* | *allele* | *symbol* |  |
| 4 | 11 | 119149355 | TATG | T | *CBL* | CBL proto-oncogene |
| 5 | 12 | 66935616 | C | T | *GRIP1* | Glutamate receptor interacting protein 1 |
| 5 | 12 | 111885299 | GCG | GAGGG | *SH2B3* | SH2B adaptor protein 3 |
| 12 | 9 | 5078360 | A | G | *JAK2* | Janus kinase 2 |
| 13 | 9 | 712156 | T | G | *KANK1* | KN motif and ankyrin repeat domains 1 |
| 13 | 9 | 732536 | T | C | *"* | *"* |
| 13 | 9 | 14720357 | C | T | *CER1* | Cerberus 1, DAN family BMP antagonist |
| 13 | 9 | 14722616 | G | A | *"* | *"* |
| 13 | 9 | 14842658 | C | G | *FREM1* | FRAS1 related extracellular matrix 1 |
| 13 | 9 | 17340864 | C | T | *CNTLN* | Centlein |
| 13 | 9 | 17486984 | C | T | *"* | *"* |
| 13 | 9 | 20953049 | A | G | *FOCAD* | Focadhesin |
| 13 | 9 | 21816758 | G | A | *MTAP* | Methylthioadenosine phosphorylase |
| 13 | 9 | 27062721 | C | T | *IFT74* | Intraflagellar transport 74 |
| 13 | 9 | 27190655 | G | A | *TEK* | TEK receptor tyrosine kinase |
| 13 | 9 | 27524364 | G | GTGTT | *IFNK* | Interferon kappa |
| 13 | 9 | 32526146 | G | A | *DDX58* | DExD/H-box helicase 58 |
| 13 | 9 | 33944383 | G | A | *UBAP2* | Ubiquitin associated protein 2 |
| 21 | 6 | 656343 | T | C | *HUS1B* | HUS1 checkpoint clamp component B |
| 21 | 6 | 7727475 | T | C | *BMP6* | Bone morphogenetic protein 6 |
| 21 | 6 | 16306751 | G | A | *ATXN1* | Ataxin 1 |
| 21 | 6 | 25850845 | C | T | *SLC17A3* | Solute carrier family 17 member 3 |
| 21 | 6 | 26091179 | C | G | *HFE* | Hemochromatosis |
| 21 | 6 | 27277051 | C | T | *POM121L2* | POM121 transmembrane nucleoporin like 2 |
| 21 | 6 | 27279774 | C | T | *"* | *"* |
| 21 | 6 | 28056646 | T | G | *ZNF165* | Zinc finger protein 165 |
| 21 | 6 | 28227436 | A | G | *NKAPL* | NFKB activating protein like |
| 21 | 6 | 28227604 | C | A | *"* | *"* |
| 21 | 6 | 28228342 | A | G | *"* | *"* |
| 21 | 6 | 28269407 | G | A | *PGBD1* | PiggyBac transposable element derived 1 |
| 21 | 6 | 28403388 | G | A | *ZSCAN23* | Zinc finger and SCAN domain containing 23 |
| 21 | 6 | 28483482 | A | C | *GPX6* | Glutathione peroxidase 6 |
| 21 | 6 | 28963248 | T | G | *ZNF311* | Zinc finger protein 311 |
| 21 | 6 | 29012712 | T | C | *OR2W1* | Olfactory receptor family 2 subfamily W member 1 |
| 21 | 6 | 29523952 | A | G | *UBD* | Ubiquitin D |
| 21 | 6 | 30880097 | T | C | *GTF2H4* | General transcription factor IIH subunit 4 |
| 21 | 6 | 31002452 | A | G | *MUC22* | Mucin 22 |
| 21 | 6 | 31002527 | A | G | *"* | *"* |
| 21 | 6 | 31084163 | A | G | *CDSN* | Corneodesmosin |
| 21 | 6 | 31929014 | A | C | *SKIV2L* | Ski2 like RNA helicase |
| 21 | 6 | 31946792 | G | A | *STK19* | Serine/threonine kinase 19 |
| 21 | 6 | 32188640 | T | C | *NOTCH4* | Notch 4 |
| 21 | 6 | 32191658 | TAGC^a^ | T | *"* | *"* |
| 21 | 6 | 32796685 | A | G | *TAP2* | Transporter 2, ATP binding cassette subfamily B member |
| 21 | 6 | 35436571 | C | T | *RPL10A* | Ribosomal protein L10a |
| 21 | 6 | 35980121 | C | T | *SLC26A8* | Solute carrier family 26 member 8 |
| 21 | 6 | 36269725 | A | G | *PNPLA1* | Patatin like phospholipase domain containing 1 |
| 21 | 6 | 36274153 | C | T | *"* | *"* |
| 21 | 6 | 41162518 | T | C | *TREML2* | Triggering receptor expressed on myeloid cells like 2 |
| 21 | 6 | 42933464 | G | A | *PEX6* | Peroxisomal biogenesis factor 6 |
| 21 | 6 | 42992825 | C | G | *RRP36* | Ribosomal RNA processing 36 |
| 21 | 19 | 1467684 | C | G | *APC2* | APC2, WNT signaling pathway regulator |
| 21 | 19 | 4294807 | G | A | *TMIGD2* | Transmembrane and immunoglobulin domain containing 2 |
| 21 | 19 | 5604875 | T | C | *SAFB2* | Scaffold attachment factor B2 |
| 21 | 19 | 7038129 | C | T | *MBD3L4* | Methyl-CpG binding domain protein 3 like 4 |
| 21 | 19 | 7755159 | A | G | *FCER2* | Fc fragment of IgE receptor II |
| 21 | 19 | 7755285 | A | G | *"* | *"* |
| 21 | 19 | 9076278 | G | A | *MUC16* | Mucin 16, cell surface associated |
| 21 | 19 | 11526765 | G | T | *RGL3* | Ral guanine nucleotide dissociation stimulator like 3 |
| 21 | 19 | 14165204 | G | C | *PALM3* | Paralemmin 3 |
| 21 | 19 | 15574917 | G | A | *RASAL3* | RAS protein activator like 3 |
| 21 | 19 | 17949108 | C | T | *JAK3* | Janus kinase 3 |
| 21 | 19 | 18562438 | C | T | *ELL* | Elongation factor for RNA polymerase II |
| 22 | 3 | 11340860 | G | A | *ATG7* | Autophagy related 7 |
| 22 | 3 | 132244508 | C | T | *DNAJC13* | DnaJ heat shock protein family (Hsp40) member C13 |

Chr, chromosome; UPD, uniparental disomy.

^a^Deletion of six AGC repeats; from 12 in the remission sample to six in the diagnostic sample.
