# Supplementary material for: Whole-exome sequencing exploration of acquired uniparental disomies in B-cell precursor acute lymphoblastic leukemia
Source: Leukemia. 2018 Jul 2;32(9):2058–62. doi: 10.1038/s41375-018-0191-0 (PMC6127080; doi:10.1038/s41375-018-0191-0)
Supplement: Supplementary file 5 — Supplementary Table 4 [file 41375_2018_191_MOESM5_ESM.docx]

**Supplementary Table 4.** The 59 candidate somatic homozygous variants in 45 genes in case 21 that could not be Sanger sequenced due to lack of DNA

| *Chr* | *Start* | *Reference* | *Variant* | *Gene* | *Gene name* |
| --- | --- | --- | --- | --- | --- |
| *No.* | *(GRCh37)* | *allele* | *allele* | *symbol* |  |
| 6 | 25862466 | C | T | *SLC17A3* | Solute carrier family 17 member 3 |
| 6 | 28264681 | G | A | *PGBD1* | PiggyBac transposable element derived 1 |
| 6 | 28403629 | T | C | *ZSCAN23* | Zinc finger and SCAN domain containing 23 |
| 6 | 30955200 | GGC | GAT | *MUC21* | Mucin 21, cell surface associated |
| 6 | 30955211 | C | T | *"* | *"* |
| 6 | 32011235 | C | T | *TNXB* | Tenascin XB |
| 6 | 32050067 | T | C | *"* | *"* |
| 6 | 33153528 | C | T | *COL11A2* | Collagen type XI alpha 2 chain |
| 6 | 35960390 | C | T | *SLC26A8* | Solute carrier family 26 member 8 |
| 6 | 42986363 | C | T | *KLHDC3* | Kelch domain containing 3 |
| 19 | 844042 | G | A | *PRTN3* | Proteinase 3 |
| 19 | 1802482 | G | A | *ATP8B3* | ATPase phospholipid transporting 8B3 |
| 19 | 2252990 | C | G | *JSRP1* | Junctional sarcoplasmic reticulum protein 1 |
| 19 | 2917287 | C | A | *ZNF57* | Zinc finger protein 57 |
| 19 | 3015692 | C | T | *TLE2* | Transducin like enhancer of split 2 |
| 19 | 4013276 | A | T | *PIAS4* | Protein inhibitor of activated STAT 4 |
| 19 | 4200156 | G | A | *ANKRD24* | Ankyrin repeat domain 24 |
| 19 | 4216910 | G | A | *"* | *"* |
| 19 | 5772950 | G | A | *CATSPERD* | Cation channel sperm associated auxiliary subunit delta |
| 19 | 6678030 | T | G | *C3* | Complement C3 |
| 19 | 7051376 | G | A | *MBD3L2* | Methyl-CpG binding domain protein 3 like 2 |
| 19 | 7535209 | G | C | *ARHGEF18* | Rho/Rac guanine nucleotide exchange factor 18 |
| 19 | 7747293 | T | G | *TRAPPC5* | Trafficking protein particle complex 5 |
| 19 | 8121369 | C | T | *CCL25* | C-C motif chemokine ligand 25 |
| 19 | 8160370 | T | C | *FBN3* | Fibrillin 3 |
| 19 | 8168545 | C | T | *"* | *"* |
| 19 | 8197865 | C | T | *"* | *"* |
| 19 | 8486884 | G | A | *MARCH2* | Membrane associated ring-CH-type finger 2 |
| 19 | 9011412 | C | T | *MUC16* | Mucin 16, cell surface associated |
| 19 | 10085054 | G | A | *COL5A3* | Collagen type V alpha 3 chain |
| 19 | 10088271 | C | G | *"* | *"* |
| 19 | 10102676 | A | G | *"* | *"* |
| 19 | 10132318 | T | C | *RDH8* | Retinol dehydrogenase 8 |
| 19 | 11313256 | G | A | *DOCK6* | Dedicator of cytokinesis 6 |
| 19 | 12384580 | T | C | *ZNF44* | Zinc finger protein 44 |
| 19 | 12541250 | T | C | *ZNF443* | Zinc finger protein 443 |
| 19 | 12541541 | CCAA | CTGC | *"* | *"* |
| 19 | 12780204 | T | C | *WDR83OS* | WD repeat domain 83 opposite strand |
| 19 | 13010520 | A | G | *GCDH* | Glutaryl-CoA dehydrogenase |
| 19 | 14029592 | C | T | *CC2D1A* | Coiled-coil and C2 domain containing 1A |
| 19 | 15198666 | T | A | *OR1I1* | Olfactory receptor family 1 subfamily I member 1 |
| 19 | 15769625 | A | G | *CYP4F3* | Cytochrome P450 family 4 subfamily F member 3 |
| 19 | 15770027 | A | T | *"* | *"* |
| 19 | 16008388 | A | C | *CYP4F2* | Cytochrome P450 family 4 subfamily F member 2 |
| 19 | 16060117 | C | A | *OR10H4* | Olfactory receptor family 10 subfamily H member 4 |
| 19 | 16060248 | A | G | *"* | *"* |
| 19 | 16060577 | T | C | *"* | *"* |
| 19 | 16060658 | A | G | *"* | *"* |
| 19 | 17000632 | G | A | *F2RL3* | F2R like thrombin or trypsin receptor 3 |
| 19 | 17396344 | G | A | *ANKLE1* | Ankyrin repeat and LEM domain containing 1 |
| 19 | 17535472 | G | A | *MVB12A* | Multivesicular body subunit 12A |
| 19 | 17638121 | G | A | *FAM129C* | Family with sequence similarity 129 member C |
| 19 | 17889044 | G | A | *FCHO1* | FCH domain only 1 |
| 19 | 18123738 | T | C | *ARRDC2* | Arrestin domain containing 2 |
| 19 | 18180413 | C | G | *IL12RB1* | Interleukin 12 receptor subunit beta 1 |
| 19 | 18180451 | A | G | *"* | *"* |
| 19 | 18186618 | T | C | *"* | *"* |
| 19 | 18280096 | G | A | *PIK3R2* | Phosphoinositide-3-kinase regulatory subunit 2 |
| 19 | 18329784 | C | T | *PDE4C* | Phosphodiesterase 4C |

Chr, chromosome.
